# Platelet-Activating Factor-Receptor Signaling Mediates Targeted Therapies-Induced Microvesicle Particles Release in Lung Cancer Cells

**DOI:** 10.3390/ijms21228517

**Published:** 2020-11-12

**Authors:** Shreepa J. Chauhan, Anita Thyagarajan, Yanfang Chen, Jeffrey B. Travers, Ravi P. Sahu

**Affiliations:** 1 Department of Pharmacology and Toxicology, Boonshoft School of Medicine Wright State University, Dayton, OH 45345, USA; Chauhan.22@wright.edu (S.J.C.); yanfang.chen@wright.edu (Y.C.); jeffrey.travers@wright.edu (J.B.T.); 2 Department of Dermatology, Wright State Physicians, Wright State University, Dayton, OH 45345, USA

**Keywords:** lung cancer, targeted therapies, platelet-activating factor-receptor, microvesicle particles

## Abstract

Microvesicle particles (MVP) secreted by a variety of cell types in response to reactive oxygen species (ROS)-generating pro-oxidative stressors have been implicated in modifying the cellular responses including the sensitivity to therapeutic agents. Our previous studies have shown that expression of a G-protein coupled, platelet-activating factor-receptor (PAFR) pathway plays critical roles in pro-oxidative stressors-mediated cancer growth and MVP release. As most therapeutic agents act as pro-oxidative stressors, the current studies were designed to determine the role of the PAFR signaling in targeted therapies (i.e., gefitinib and erlotinib)-mediated MVP release and underlying mechanisms using PAFR-expressing human A549 and H1299 non-small cell lung cancer (NSCLC) cell lines. Our studies demonstrate that both gefitinib and erlotinib generate ROS in a dose-dependent manner in a process blocked by antioxidant and PAFR antagonist, verifying their pro-oxidative stressor’s ability, and the role of the PAFR in this effect. We observed that these targeted therapies induce MVP release in a dose- and time-dependent manner, similar to a PAFR-agonist, carbamoyl-PAF (CPAF), and PAFR-independent agonist, phorbol myristate acetate (PMA), used as positive controls. To confirm the PAFR dependency, we demonstrate that siRNA-mediated PAFR knockdown or PAFR antagonist significantly blocked only targeted therapies- and CPAF-mediated but not PMA-induced MVP release. The use of pharmacologic inhibitor strategy suggested the involvement of the lipid ceramide-generating enzyme, acid sphingomyelinase (aSMase) in MVP biogenesis, and observed that regardless of the stimuli used, aSMase inhibition significantly blocked MVP release. As mitogen-activated protein kinase (MAPK; ERK1/2 and p38) pathways crosstalk with PAFR, their inhibition also significantly attenuated targeted therapies-mediated MVP release. These findings indicate that PAFR signaling could be targeted to modify cellular responses of targeted therapies in lung cancer cells.

## 1. Introduction

Lung cancer is currently the second most prevalent type of cancer in both men and women in the United States, which continues to be a major health and socioeconomic issues [1,2,3]. Several risk factors have been known to be associated with lung carcinogenesis, including environmental factors and pollutants such as smoking and arsenic exposure, etc., as well as genetic factors [4,5,6]. While lung cancer cases have also been documented in non-smoking patients, smoking remains the most common contributing factor as long-term exposure to smoking has been shown to induce dysregulation and disruption in healthy lungs [7,8,9]. Of two main types, non-small cell lung carcinoma (NSCLC) accounts for the majority of the cases diagnosed compared to a less prevent small cell lung carcinoma (SCLC) [10,11]. Though significant achievements have been made in molecular pathogenesis and cellular signaling pathways involved in the development of lung cancer, the mechanisms leading to immunoevasion or the induction of tumor resistance to the known therapeutic regimens are yet to be fully explored [12,13,14]. Depending upon the lung cancer stages, the treatment options include surgical resection, chemotherapy, radiation therapy, targeted and immune-based approaches, as well as a combination of two or more therapeutic agents [15,16,17,18,19].

Among targeted therapies, receptor tyrosine kinase inhibitors (TKIs) including gefitinib and erlotinib have been used to treat NSCLC patients [20,21,22]. The primary mechanism of TKIs is to target tumor cells harboring activating mutations in the epidermal growth factor receptor (EGFR), a receptor tyrosine kinase of the ErbB family, which plays important roles in sustained tumor cell proliferation [20,21,22]. However, EGFR-independent mechanisms of action of these TKIs have also been documented, of which the most notable is the induction of oxidative stress, attributable to their pro-oxidative stressors ability to generate reactive oxygen species (ROS) [23,24,25]. While ROS generation remains one of the common mechanisms of cancer therapies including TKIs to induce cytotoxic effects, this ROS-mediated induction of oxidative stress has also been implicated in the development of tumor resistance mechanisms to such therapeutic options [26,27,28].

Studies, including ours, have shown that ROS-generating pro-oxidative stressors such as chemotherapeutic agents and radiation therapy produce oxidized lipid mediator glycerophosphocholines (ox-GPCs) as a bystander effect [29,30,31]. Many of these ox-GPCs exhibit platelet-activating factor (PAF)-like agonistic activity and bind to a G-protein coupled, PAF-receptor (*Ptafr*; PAFR), expressed on a variety of cell types including tumor cells [29,30,31]. Relevant to lung cancer, our previous studies have shown that exposure to cigarette smoke generates PAF agonists in a PAFR-dependent manner that induce systemic immunosuppression in a process blocked by antioxidants, cyclooxygenase types 2 (COX-2) inhibitors, and PAF-metabolizing enzyme PAF-acetyl hydrolase (PAF-AH), as well as depleting antibodies against immunosuppressive regulatory T cells (Tregs) [32]. We have also shown that the systemic administration of a known PAFR-agonist, carbamoyl-PAF (CPAF) can augment the growth of Lewis lung carcinoma (LLC1) tumors and its metastatic ability in a PAFR-dependent manner [33]. However, the role of the PAFR signaling in modulating targeted therapy effects in lung cancer models has not been studied. Given that these PAF agonists are metabolically labile and upon generation get readily metabolized by PAF-AH, our recent studies have defined the critical roles of a subpopulation of extracellular vesicles known as microvesicles or microvesicle particles as a novel mechanism by which these potent lipids are not only protected but circulated to exert local as well as delayed systemic effects [34,35,36].

Extracellular vesicles (EVs) are released from a wide variety of cell types and serve to mediate cell-to-cell communications, both in physiological and pathological conditions. These EVs mainly comprise of three types, which differ particularly in their sizes, for example, exosomes (size ~30–100 nm), microvesicle particles (MVP, size ~100–1000 nm), and apoptotic bodies (size >1000 nm) [35,36], their contents, and mechanism of formation. In as much as PAFR signaling plays critical roles ranging from acute pro-inflammatory to delayed systemic immunosuppression and augmentation of tumor growth as well as limiting the efficacy of therapeutic agents [29,30,31,32,33], the current studies were sought to determine its relevance in targeted therapies-mediated MVP release and define the underlying mechanisms in lung cancer cells. Overall, our studies demonstrate that targeted therapies act as pro-oxidative stressors, and induce MVP release in a PAFR-dependent manner via mechanisms involving acid sphingomyelinase (aSMase) enzyme, and mitogen-activated protein kinase (MAPK) pathway.

## 2. Results

### 2.1. Exposure to Gefitinib and Erlotinib Generates ROS

As several human malignancies including lung cancer express functional PAFR [29,37,38,39], which upon the exposure to pro-oxidative stressors including chemotherapeutic agents and radiation therapy can produce PAF agonists [29,30,31], our first studies verified the pro-oxidative stressors ability of targeted therapies to generate ROS. For this, we took advantage of a recent study, which identified that some NSCLC cell lines including A549 and H1299 express functional PAFR [38]. Besides, these cell lines also harbor wild-type EGFR, and have been used in studies with gefitinib and/or erlotinib [28,40,41,42,43].

**Figure 1:**
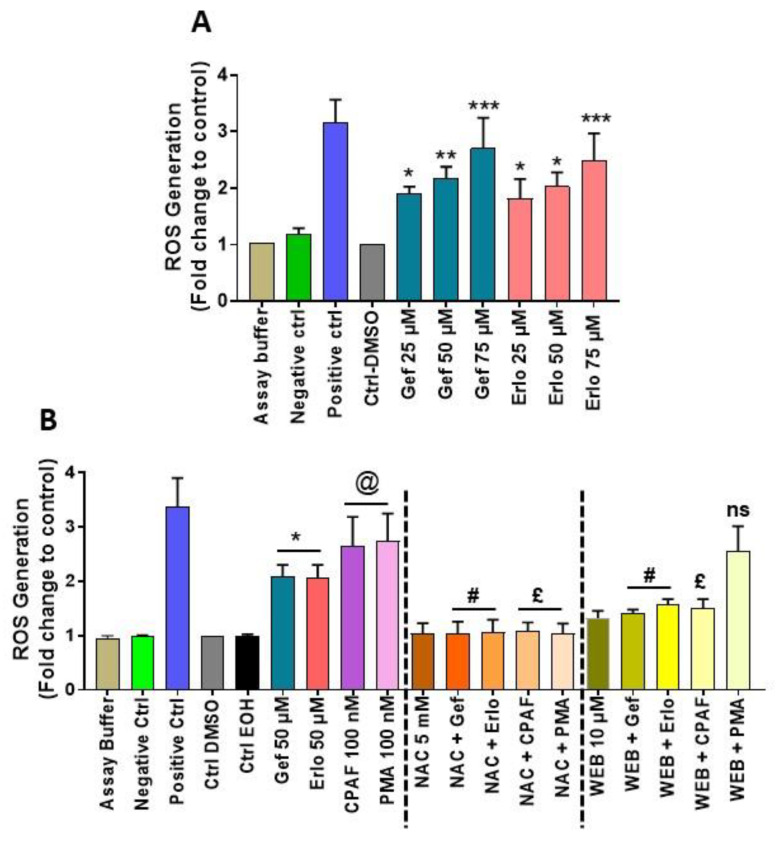
Effects of gefitinib and erlotinib on reactive oxygen species (ROS) generation. (**A**) A549 cells were treated with 0.1% dimethyl sulfoxide (DMSO) as vehicle control, and gefitinib (Gef) or erlotinib (Erlo) at 25, 50, and 75 µM concentrations. The cells were also treated with assay buffer, negative and positive controls from the manufacturer’s kit. After 30 min of incubation, ROS generation was measured using a Synergy microplate reader. Data are expressed as mean ± SE of three independent experiments and presented as ROS generation (Fold change to control) versus treatment groups. Statistically significant differences were observed between Ctrl-DMSO and Gef or Erlo at various doses (* = *p* < 0.05, ** = *p* < 0.01, *** = *p* < 0.001). (**B**) A549 cells were pretreated with N-acetylcysteine (NAC; 5 mM) or WEB2086 (10 µM) for 1 h followed by the treatments with or without Gef, Erlo, carbamoyl-PAF (CPAF), or phorbol myristate acetate (PMA), and after 30 min of incubation, ROS generation was measured. Data are expressed as mean ± SE of five independent experiments and presented similar to Figure 1A. Statistically significant differences were observed between Ctrl-DMSO and Gef or Erlo (* = *p* < 0.05); Ctrl-ethanol (EOH) vs. CPAF or PMA (@ = *p* < 0.05); Gef vs. NAC + Gef or Erlo vs. NAC + Erlo (# = *p* < 0.01); Gef vs. WEB + Gef or Erlo vs. WEB + Erlo (£ = *p* < 0.05); CPAF vs. NAC + CPAF or PMA vs. NAC + PMA (# = *p* < 0.05); and CPAF vs. WEB + CPAF (£ = *p* < 0.05). ns denotes non-significant change between PMA and WEB + PMA.

To measure ROS generation, we treated A549 cells (used as a model) with or without gefitinib and erlotinib at various doses (25, 50, and 75 µM), similar to as previously reported [28,40,41,42]. Since ROS generation is an earlier event, we assessed its generation after 30 min of incubation along with assay buffer and negative and positive controls via measuring DCFDA fluorescence as per the kit’s protocol. We observed that both these agents generate ROS in a dose-dependent manner, which verified that these targeted therapies act as pro-oxidative stressors (Figure 1A), as also reported previously [24,25]. To confirm the roles of ROS, and PAFR on targeted therapies-induced ROS generation, A549 cells were pretreated with antioxidant, N-acetylcysteine (NAC), and PAFR antagonist, WEB2086 compounds. Following 1 h of incubation, cells were treated with or without gefitinib and erlotinib at an optimum dose of 50 µM, along with PAFR agonist, CPAF, and PAFR-independent agonist, phorbol myristate acetate (PMA) used as positive controls. We also used two different solvents (i.e., DMSO and ethanol [EOH]) as negative controls to precisely rule out their effects on gefitinib and erlotinib (dissolved in DMSO) versus CPAF and PMA (dissolved in EOH)-induced ROS generation. After 30 min of incubation, ROS generation was assessed. Our studies demonstrate that targeted therapies-induced ROS generation is significantly reduced by NAC and WEB2086 compounds, similar to as observed by CPAF treatment (Figure 1B). However, PMA-induced ROS generation is only significantly attenuated by NAC but not by WEB2086 (Figure 1B). These studies confirmed that targeted therapies induce ROS generation, and suggest the role of the PAFR signaling in mediating this effect.

### 2.2. Gefitinib and Erlotinib Treatments Induce MVP Release from NSCLC Cell Lines in a Time- and Dose-Dependent Manner

**Figure 2:**
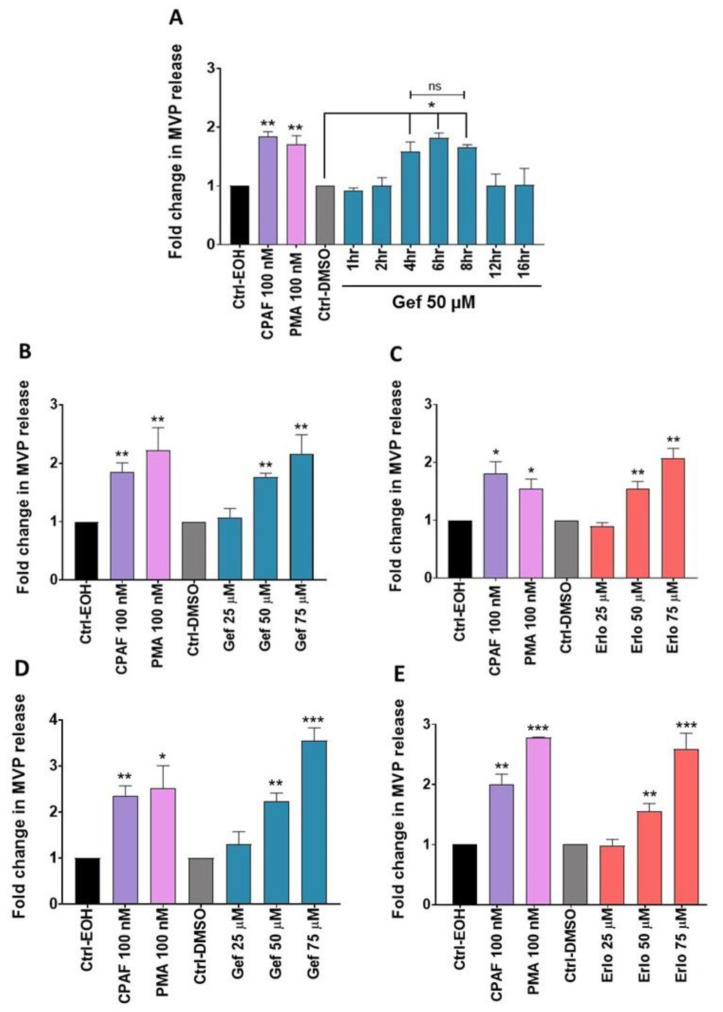
Time and dose-response assessments of gefitinib and erlotinib on microvesicle particles (MVP) release. (**A**) A549 cells were treated with 0.1% EOH or DMSO as vehicle controls, 100 nM CPAF or PMA as positive controls, or 50 µM Gef. After incubation at the given time points, MVPs were isolated and analyzed. (**B**,**C**) Similarly, A549 cells were treated with vehicle controls, CPAF, PMA, and Gef or Erlo at 25, 50, and 75 µM doses. (**D**,**E**) Similar experiments as mentioned for A549 cells were performed with H1299 cells. After 4 h of incubation, MVPs were isolated and analyzed. Data are representative of mean ± SE of three independent experiments, normalized per 1 × 10^6^ cells. Statistically significant differences were observed between Ctrl-EOH and CPAF or PMA (* = *p* < 0.05, ** = *p* < 0.01); Ctrl-DMSO and Gef or Erlo at various doses (* = *p* < 0.05, ** = *p* < 0.01, *** = *p* < 0.001); ns denotes non-significant changes between the analyzed groups.

Given that gefitinib and erlotinib generate ROS in a process blocked by antioxidant and PAFR antagonist (Figure 1), and our previous reports demonstrating that pro-oxidative stressors induce MVP release in a PAFR-dependent manner [34,35,36], we tested our working hypothesis if these targeted therapies can induce MVP release in lung cancer cells. It should be noted that depending upon the nature of the stimuli or the cell types, the secretion of MVPs has been shown to be time and/or concentration-dependent [44]. Thus, our first studies evaluated the time-dependent response of gefitinib (used as a drug model) on MVP release from the A549 cell line using CPAF and PMA as positive controls, as per our previous reports [34,35,36]. We observed that gefitinib induces MVP release in a time-dependent manner that significantly peaks between 4 to 8 h, yet no differences in MVP release were noted between these time points (Figure 2A). Thus, we used a 4-hours’ time point to evaluate the dose-response effects of these targeted therapies along with appropriate controls on MVP release from A549 and H1299 cell lines. Our studies demonstrate that gefitinib and erlotinib induce MVP release from both A549 (Figure 2B,C) and H1299 (Figure 2D,E) cell lines in a dose-dependent manner.

### 2.3. Effect of Gefitinib and Erlotinib Treatments on Apoptosis Induction

**Figure 3:**
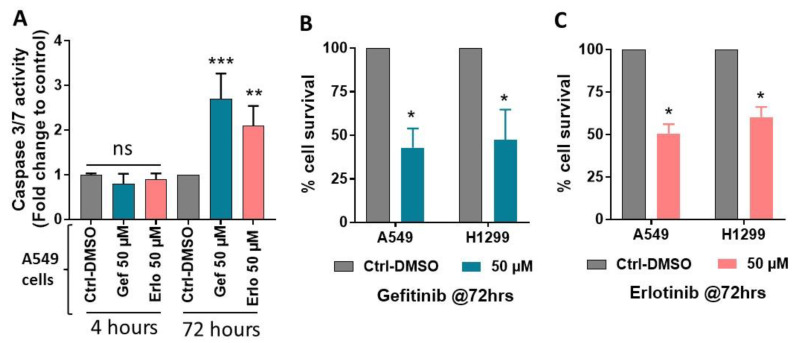
Effects of gefitinib and erlotinib on apoptosis induction and survival of NSCLC cell lines. (**A**) A549 cells were treated with 0.1% DMSO as vehicle control and Gef or Erlo at 50 µM dose. After 4 h and 72 h of incubation, apoptosis induction was assessed by quantitative caspase 3/7 activity assay. Similarly, (**B**) A549 and (**C**) H1299 cell lines were treated with vehicle control, and Gef or Erlo and after 72 h, cell survival was assessed by sulforhodamine B (SRB) assay. Data are expressed as mean ± SE of three independent experiments and represented as (**A**) Caspase 3/7 activity (Fold change to control) over treatment groups along with time points, and (**B**,**C**) % cell survival over treatment groups with their respective cell lines. Statistically significant differences were observed between Ctrl-DMSO and Gef or Erlo with their respective time points or cell lines used (* = *p* < 0.05, ** = *p* < 0.01, *** = *p* < 0.001); ns indicates non-significant changes between the analyzed groups.

To address if gefitinib and erlotinib specifically induce MVP, or can also stimulate the secretion of other extracellular vesicles such as apoptotic bodies, we assessed apoptosis induction at the same experimental settings that were used to measure MVP release. For this, A549 cells were treated with or without gefitinib and erlotinib at an optimal dose of 50 µM, and after 4 h of incubation, apoptosis induction was assessed by quantitative caspase 3/7 activity assay. We did not observe apoptosis induction by these targeted therapies as compared to vehicle-treated cells at 4 hours’ time point (Figure 3A). However, significantly increased apoptosis was detected following gefitinib and erlotinib treatments compared to the vehicle-treated cells at 72 hours’ time point (Figure 3A). We also noticed significantly decreased % cell survival by gefitinib and erlotinib treatments compared to the vehicle-treated A549 and H1299 cell lines at 72 hours’ time point (Figure 3B,C), as also reported previously [28,40,43]. These data indicate that targeted therapies-mediated MVP release is an earlier event as also confirmed by the findings that MVP release peaks between 4 to 8 hours’ time point, and then declines at 12 hours’ time point (Figure 2A). These data also indicate that apoptosis induction or cell growth inhibition occurs at a later time point that mediates the cytotoxic effects of gefitinib and erlotinib.

### 2.4. Blockade of the PAFR Attenuates Erlotinib and Gefitinib-Induced MVP Release

**Figure 4:**
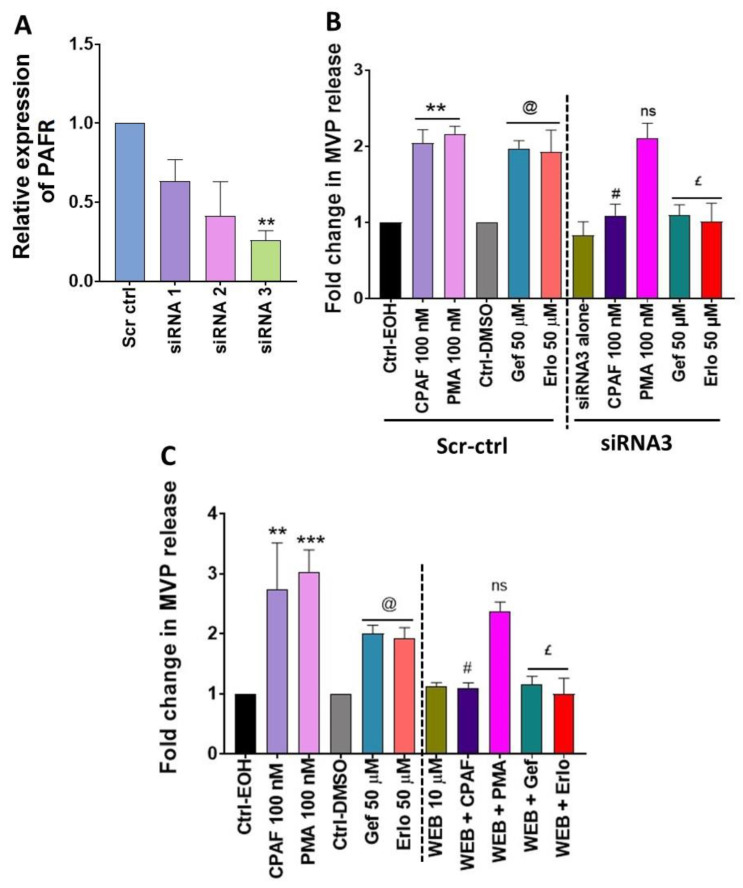
Effects of the PAFR-specific siRNA and PAFR antagonist on gefitinib and erlotinib-induced MVP release. (**A**) A549 cells were transfected with scrambled siRNA (i.e., Scr-ctrl) for control or three separate clones of PAFR siRNA and after 48 h, the PAFR knockdown efficiency was evaluated by qPCR assay. (**B**) A549 cells were transfected with Scr-ctrl or PAFR siRNA clone 3 (i.e, siRNA3) and after 48 h treated with 0.1% EOH or DMSO as vehicle controls, 100 nM CPAF or PMA as positive controls, and 50 µM Gef or Erlo. (**C**) A549 cells were pretreated with PAFR antagonist, WEB2086 (10 µM, 1 h) followed by the treatments with or without CPAF, PMA, Gef, or Erlo at the given doses. After 4 h of incubation, MVPs were isolated and analyzed. Data are mean ± SE of three independent experiments, normalized per 1 × 10^6^ cells, and represented as (**A**) Relative expression of PAFR over Scr-ctrl and 3 separate clones of PAFR siRNA, and (**B**,**C**) Fold change in MVP release over various treatment groups. The statistically significant differences were observed between (**A**) Scr-ctrl and siRNA3 (** = *p* < 0.01), (**B**) Ctrl-EOH and CPAF or PMA (** = *p* < 0.01); ctrl-DMSO and Gef or Erlo (@ = *p* < 0.01); CPAF vs. siRNA3 + CPAF (# = *p* < 0.01); PMA vs. siRNA3 + PMA (ns); Gef vs. siRNA3 + Gef, and Erlo vs. siRNA3 + Erlo (£ = *p* < 0.01), (**C**) Ctrl-EOH and CPAF or PMA (** = *p* < 0.01, *** = *p* < 0.001); Ctrl-DMSO and Gef or Erlo (@ = *p* < 0.01); CPAF vs. WEB + CPAF (# = *p* < 0.01); PMA vs. WEB + PMA (ns); Gef vs. WEB + Gef or Erlo vs. WEB + Erlo (£ = *p* < 0.01); ns indicates non-significant changes between the analyzed groups.

To confirm if PAFR signaling is essential in gefitinib and erlotinib-induced MVP release, we adopted two separate approaches: (1) knocking down PAFR expression via PAFR specific siRNA; and (2) using a specific PAFR antagonist, WEB2086. Given the relatively same expression of the PAFR protein in A549 and H1299 cell lines [38] with similar dose-response effects of gefitinib and erlotinib on MVP release (~2–3 folds; Figure 2B,E), or inhibition of % cell survival (~50%; Figure 3B,C), we used A549 cells for our next studies. To optimize PAFR gene silencing, A549 cells were transfected with scrambled siRNA for control (i.e., Scr-ctrl) or three separate clones of PAFR siRNA, and the knockdown efficiency was evaluated by quantitative RT-PCR (qPCR) assay. We found that all PAFR siRNA clones decreased its mRNA expression with significant knockdown was detected with clone 3 (i.e., siRNA3) compared to Scr-ctrl (Figure 4A). In the next studies, A549 cells were transfected with either Scr-ctrl or siRNA3 followed by the treatments with or without CPAF, PMA, gefitinib, or erlotinib, and MVP release was quantified. In separate experiments, we tested the effect of WEB2086 pretreatment on CPAF, PMA, gefitinib, or erlotinib-mediated MVP secretion. Our studies demonstrate that both the knockdown or blockade of the PAFR resulted in significantly decreased MVP release induced by CPAF, gefitinib, and erlotinib as compared to these treatments alone (Figure 4B,C). However, neither PAFR siRNA3 nor WEB2086 exerted any effects on PMA-induced MVP release when compared to PMA-alone or the vehicle control treatments (Figure 4B,C). These findings indicate the necessity of the PAFR signaling in targeted therapies-induced MVP release.

### 2.5. Inhibition of aSMase Blocks MVP Release

**Figure 5:**
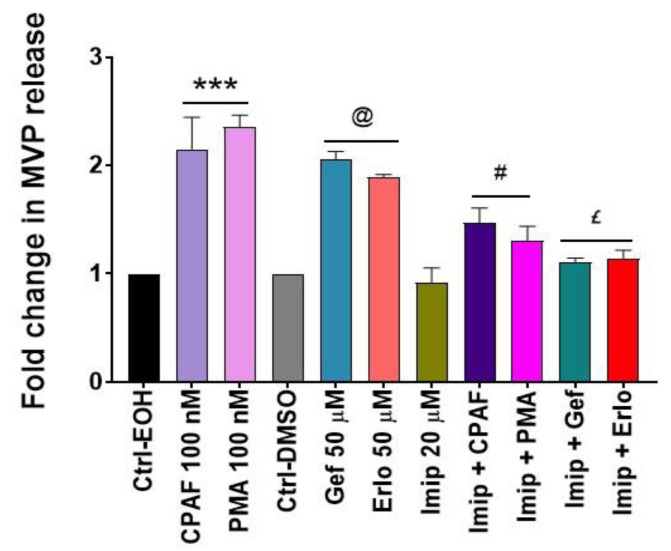
Acid sphingomyelinase (aSMase) inhibition abrogates gefitinib-induced MVP release. A549 cells were pretreated with aSMase inhibitor, imipramine (Imip; 20 µM, 1 h) followed by the treatments with 0.1% EOH or DMSO as vehicle controls, 100 nM CPAF or PMA as positive controls, and 50 µM Gef or Erlo. After 4 h of incubation, MVPs were isolated and quantified. Data are mean ± SE from three independent experiments, normalized per 1 × 10^6^ cells, and represented as Fold change in MVP release over various treatment groups. Statistically significant differences were observed between Ctrl-EOH and CPAF or PMA (*** = *p* < 0.001); ctrl-DMSO and Gef or Erlo (@ = *p* < 0.001); CPAF vs. Imip + CPAF or PMA vs. Imip + PMA (# = *p* < 0.05); Gef vs. Imip + Gef or Erlo vs. Imip + Erlo (£ = *p* < 0.01).

The biogenesis of MVP involves activation of the aSMase pathway, and previous reports including ours have shown that the inhibition of aSMase blocks MVP release regardless of the stimuli used [34,35,36,45,46,47]. To that end, our next studies tested the effect of a specific pharmacologic inhibitor of aSMase (i.e., imipramine) on targeted therapies-induced MVP release. The A549 cells were pretreated with imipramine for 1 h followed by the treatments with or without CPAF, PMA, gefitinib, or erlotinib and MVP release was quantified. We observed that imipramine (Imip) treatment not only significantly blocked CPAF and PMA-mediated but also gefitinib and erlotinib-induced MVP release compared to these treatments alone (Figure 5), confirming the role of aSMase in MVP release.

### 2.6. MAPK Pathway Mediates Erlotinib and Gefitinib-Mediated MVP Release

**Figure 6:**
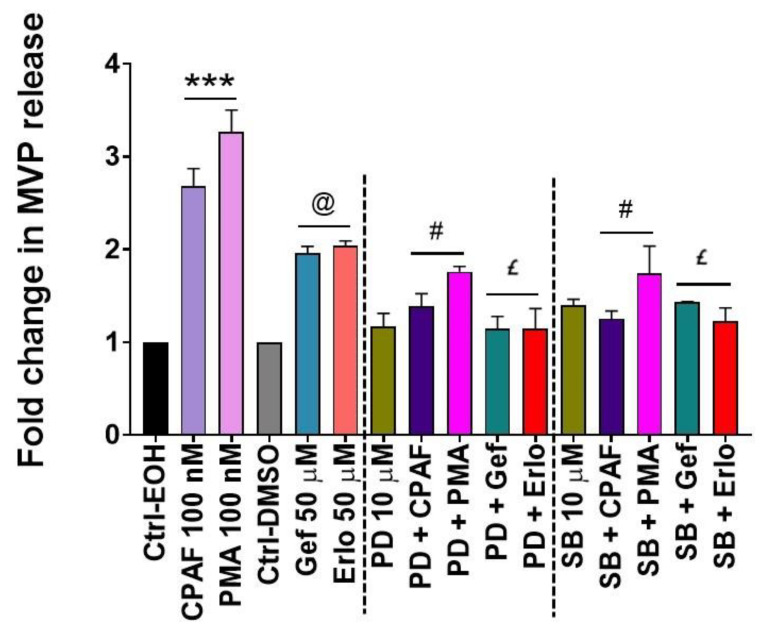
Effects of the extracellular signal-regulated kinase (ERK1/2) and p38-MAPK inhibitors on gefitinib and erlotinib-induced MVP release. A549 cells were pretreated with PD98059 and SB202190 (10 µM, 1 h) followed by the treatments with 0.1% EOH or DMSO as negative controls, 100 nM CPAF or PMA as positive controls, and 50 µM Gef or Erlo. After 4 h of incubation, we isolated and analyzed MVP secretion. Data are mean ± SE from three independent experiments, normalized per 1 × 10^6^ cells, and represented as Fold change in MVP release over various treatment groups. Statistically significant differences were observed between Ctrl-EOH and CPAF or PMA (*** = *p* < 0.001); Ctrl-DMSO and Gef or Erlo (@ = *p* < 0.01); CPAF vs. PD + CPAF or SB + CPAF (# = *p* < 0.001); PMA vs. PD + PMA or SB + PMA (# = *p* < 0.001); Gef vs. PD + Gef or SB + Gef, and Erlo vs. PD + Erlo or SB + Erlo (£ = *p* < 0.05).

**Figure 7:**
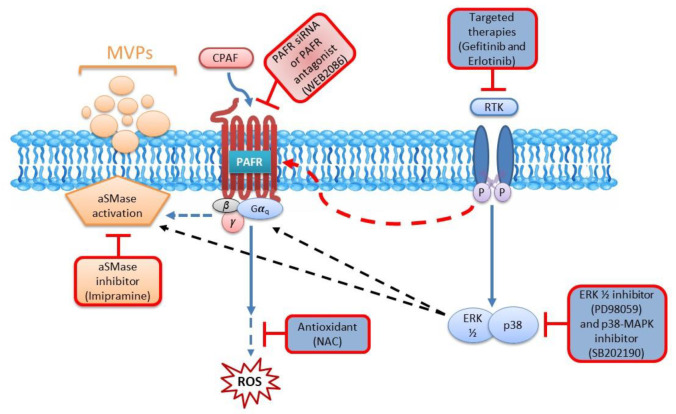
Schematic representation of a working model of PAFR-dependent targeted therapies-induced MVP release. In this model, targeted therapies activate the PAFR likely via the production of PAF agonists, similar to CPAF, which generates ROS in a process blocked by PAFR antagonist and antioxidant. The PAFR activation also stimulates aSMase, which involves ERK1/2 and p38-MAPK pathways resulting in MVPs formation and release in a process blocked by PAFR siRNA and PAFR antagonist, aSMase inhibitor, and ERK1/2 and p38-MAPK inhibitors.

Studies, including ours, have shown that the MAPK pathway, particularly extracellular signal-regulated kinase (ERK1/2) and p38-MAPK mediate not only PAFR and chemotherapy-induced effects, but also aSMase-mediated MVP biogenesis and secretion [34,48]. To that end, our next studies examined the effects of blocking ERK1/2 and p38-MAPK pathways using their specific inhibitors (i.e., PD90859 [PD] and SB202190 [SB] compounds), respectively. For this, A549 cells were pretreated with PD or SB compounds for 1 h followed by the treatments with or without CPAF, PMA, gefitinib, or erlotinib, and MVP release was quantified. We demonstrate that both ERK1/2 and p38-MAPK inhibition significantly attenuated CPAF, gefitinib, and erlotinib-induced MVP secretion compared to these treatments alone (Figure 6). Interestingly, PMA-induced MVP release was also significantly blocked by PD and SB compounds (Figure 6). These findings are not entirely surprising given that the PMA-induced phospholipase C (PLC) activation interacts with the phosphoinositide 3-kinase (PI3K) pathway, which indicates the crosstalk of the MAPK signaling with both PI3K and PAFR pathways [49,50]. The schematic representation of our working model is shown in Figure 7.

## 3. Discussion

PAF is a potent phospholipid mediator, implicated in several pathological as well as inflammatory conditions, which mediates its effects via binding to a widely-expressed single seven-transmembrane G protein-coupled PAFR [32,51,52,53]. Relevant to cancer, growing evidence has demonstrated the potential role of the PAFR signaling in modulating the in vitro and in vivo growth of various tumor types in response to ROS-generating pro-oxidative stressors including therapeutic agents [29,30,31,54,55,56]. In in vitro models, PAFR-expressing tumor cells generate more PAF agonists in response to chemotherapeutic agents and radiation therapy via activating tumoral PAFR, which has been shown to induce a prosurvival response to such therapeutic agents [29,30,31]. To that end, multiple studies have tested the abilities of PAFR antagonists to control the in vitro cell proliferation, or in vivo tumor progression [29,54,55]. Importantly, our studies have shown that in the experimental murine melanoma model, host PAFR activation can augment the growth of tumor xenografts or impede the anti-tumoral immune response of therapeutic agents via mechanisms involving COX-2-dependent upregulation of Tregs [30,31,56].

Particularly to the lung cancer model, we have shown that activation of the host PAFR (via studies using PAFR-expressing wild type and PAFR-deficient mice) not only augments the growth but also the metastatic ability of murine Lewis lung carcinoma (LLC1) cells [33]. Notably, Chen and colleagues have also demonstrated that PAFR activation enhances the in vitro cell proliferation, as well as in vivo growth of NSCLC via involving STAT3 signaling [38]. Importantly, high tumoral PAFR expression has also been found to be positively correlated with increased tumor invasiveness and tumor stages as well as the decreased survival probability of NSCLC patients [38]. As lung cancer remains one of the difficult malignancies to treat, despite the advancements in the treatment modalities including the targeted therapy approaches, studies have been directed towards deciphering the mechanisms associated with modulating the sensitivity or efficacy of therapeutic agents. Given that ROS-generating pro-oxidative stressors can produce oxidized PAF agonists from a variety of PAFR-expressing cell types, which can then travel via the MVP to exert local, as well as systemic effects [34,35,36], the current studies were designed to test our hypothesis if PAFR signaling can modulate targeted therapy-induced MVP release from lung cancer cells, and then define the underlying mechanisms.

Given a recent report demonstrating that some human NSCLC cell lines express PAFR and that A549 and H1299 cells have similar protein expression of PAFR [38], and also harbor wild type EGFR [28], we used them as models. Our first studies verified that gefitinib and erlotinib in addition to their primary mechanism of targeting tumor cells harboring mutated forms of EGFR [20,21,22] also act as pro-oxidative stressors [23,24,25]. We demonstrate that these targeted therapies generate ROS in a dose-dependent manner, as also previously reported [24,25]. To confirm the roles of ROS, and PAFR on targeted therapies-induced ROS generation, our studies evaluated the effects of antioxidant, NAC and PAFR antagonist, WEB2086 and demonstrated that both these compounds significantly attenuate gefitinib and erlotinib-induced ROS generation, similar to as observed by CPAF, used as a positive control [57]. However, PMA-induced ROS generation, used as another control [58,59,60] is blocked only by NAC but not by the WEB2086 compound. While NAC is a known ROS quencher, the WEB2086 effect is via blocking PAFR, indicating that targeted therapies-induced ROS generation is mediated via the activation of the PAFR pathway.

Given that these targeted therapies generate ROS, its potential role in exosomes and MVP release has been directly or indirectly evidenced in modifying the cellular responses, or the efficacy of targeted and conventional anticancer agents in model systems including cancer cells. While ROS are involved in the production of MVPs via its ability to induce oxidative stress, MVPs shed by cell types including tumor cells have also been shown to exhibit altered redox balances with elevated ROS levels, and that the number of MVPs are further affected by oxidative conditions such as those induced by ROS-generating pro-oxidative stressors [34,35,61]. In addition, Li and colleagues have demonstrated that exosomes-derived from gefitinib-treated EGFR-mutant human NSCLC PC9 cell line decrease the antitumor effects of cisplatin, and that pharmacological inhibition of exosomes secretion resulted in a modest synergistic effect of cisplatin and gefitinib combination [62]. Along similar lines, exosomes and MVPs inhibitors have been shown to augment the cytotoxic efficacy of a chemotherapy drug 5-fluorouracil (5-FU) against human prostate cancer PC3 and breast cancer MCF-7 cell lines compared to 5-FU alone treatment [63]. However, there is no direct evidence of the PAFR involvement in targeted therapies-induced MVP release. To that end, our next studies evaluated the time and dose responses of gefitinib on MVP release from NSCLC cell lines using CPAF and PMA as positive controls. We demonstrate that exposure to gefitinib induces MVP release in a time and dose-dependent manner. Similar dose-dependent effects of erlotinib were also observed on MVP release.

While several comprehensive studies have shown that the crosstalk between various signaling pathways mediates the cytotoxic (e.g., apoptotic) effects of anti-cancer drugs, the cell-to-cell communication by extracellular vesicles such as MVP and exosomes plays vital roles in diverse biological, and pathological processes including cancer growth and metastasis, as well as modulating the sensitivity of therapeutic agents [44,45,64,65]. To evaluate if gefitinib and erlotinib at experimental settings used to measure MVP release, can also induce apoptosis, we analyzed caspase 3/7 activity. We observed that treatments of these targeted therapies did not induce apoptosis as compared to the vehicle control treatment. However, at a later time point, we detected significantly increased apoptosis and decreased cell survival by gefitinib and erlotinib as compared to the vehicle control treatment, similar to as previously reported [28]. These findings indicate that MVP release is an earlier event, which has been implicated in modulating cancer growth, sensitivity, or the responses of anti-cancer agents [64,65,66] and that cancer growth inhibition or apoptosis induction occurs at a later time point(s) which mediate their cytotoxic effects [28,40,41,43]. Notably, MVP release during the pathological states including from tumor cells has also been implicated as potential biomarkers to predict disease stages/conditions [66,67]. Importantly, since tumor-derived exosomes and MVPs exacerbate the tumor microenvironment, multiple studies have also suggested that removal of such vesicles could be exploited to sensitize tumor cells to chemotherapy or immunotherapy approaches, or could be used as a vehicle for drug delivery [68,69,70].

The current studies are significant given the findings of another report demonstrating that circulating MVP derived from the lung cancer patients enhanced tumor angiogenesis in in vitro HUVEC cell model [71]. Besides, these results are compatible with our group’s previous reports demonstrating that exposure to UVB radiation or thermal burn injury induces MVP release from PAFR-expressing epidermal HaCaT, and KBP cell lines in a dose-dependent manner [35,36]. Moreover, another study demonstrated that gemcitabine treatment induces MVP release significantly from PAFR-expressing cell line-PANC1 but not from PAFR-deficient Hs766T cell line [34]. The translational relevance of these studies is supported by our published findings demonstrating that MVP derived from chemotherapy-treated PAFR-expressing cells contain PAF agonists [34]. These separate lines of evidence are in agreement with our current studies indicating that ROS-generating pro-oxidative stressors stimulate MVP secretion in a PAFR-dependent manner. Moreover, the data demonstrating that the blockade of PAFR activation either by PAFR antagonist or PAFR specific siRNA approach only attenuated MVP release via CPAF and targeted therapy but not by PMA confirmed the absolute requirement of the PAFR in mediating targeted therapy-induced MVP release from NSCLC cell lines.

Since the biosynthesis and release of MVP in response to multiple stimuli are aSMase-dependent [34,35,36,45,46,47,48], and that aSMase inhibition by imipramine has been used as a tool to block aSMase-mediated MVP generation [46,47]. Consistent with this notion, our studies have observed that imipramine not only blocked CPAF-induced, but also PMA, as well as gefitinib and erlotinib-mediated MVP release, confirming the role of aSMase in MVP release. In addition to the primary mechanism (i.e., targeting activating mutations in EGFR), several other targets of tyrosine kinase inhibitors including MAPK, and PI3K/AKT have been identified in NSCLC models [24,25,26,27,28,40]. Notably, the MAPK (i.e., ERK1/2 and p38) pathways have also been shown to play important roles in aSMase-dependent MVP generation, and mediating the effects of PAFR signaling in pro-oxidative stressors-induced local and systemic responses [34,36,48,72]. To verify the roles of ERK1/2 and p38 pathways in MVP release, we used their specific inhibitors and found that the inhibition of both ERK1/2 and p38 pathways resulted in significantly reduced MVP secretion mediated by CPAF, erlotinib, and gefitinib compared to these treatments alone. However, these inhibitors also blocked the PMA-induced MVP release. These findings indicate that the activation of both the PAFR and MAPK pathways are involved in mediating gefitinib and erlotinib-induced aSMase-dependent MVP release, and also supported by the studies demonstrating that MAPK crosstalks with PAFR pathway [48]. Consistent with this notion that tyrosine kinase inhibitors interact with PAFR, we have found that treatment of PAFR-positive KBP cells with gefitinib and erlotinib and results in IL-8 release, which is not found in PAFR-negative KBM cells (a separate project, data not shown).

Overall, these studies indicate the potential role of the PAFR signaling in targeted therapies-mediated MVP release, and that these observed effects are mediated via an interplay between the PAFR and MAPK pathways. Importantly, our studies are of high significance as these provide the rationale of exploring the translational relevance of this PAFR signaling targeted therapies-induced MVP release, which could have cellular and systemic effects in lung cancer.

## 4. Materials and Methods

### 4.1. Reagents

The culture media was purchased from GE Healthcare Biosciences (Marlborough, MA, USA). The PAF-R agonist (CPAF), PAF-R antagonist (WEB2086), PAF-R siRNA, erlotinib, gefitinib, ERK1/2, and p38 inhibitors, and imipramine were purchased from Cayman Chemicals Co. (Ann Arbor, MI, USA). Cell culture media, fetal bovine serum (FBS) was from Corning (Corning, NY, USA), antibiotic-antimycotic was from Gibco (Gaithersburg, MD, USA), and penicillin-streptomycin was purchased from Hyclone (Logan, UT, USA). The RNA extraction kit was purchased from Invitrogen Life Technologies (Carlsbad, CA, USA). PAFR siRNAs (FlexiTube) were from Qiagen (Germantown, MD, USA) and lipofectamine 3000 was from Invitrogen (Thermo Fisher Scientific, Carlsbad, CA, USA). The PAF-R, GAPDH primers, high-capacity cDNA Reverse Transcription kit, SYBR green, and PCR reagents were purchased from Applied Biosystems (Carlsbad, CA, USA). The ROS detection assay kit was from BioVision (BioVision Incorporated (Milpitas, CA, USA), and the Caspase-Glo 3/7 Assay kit for the measurement of apoptosis induction was from Promega Corporation (Madison, WI, USA).

### 4.2. Cell Culture

Human non-small cell lung cancer (NSCLC) cell lines A549 and H1299 (a kind gift from Dr. Weiwen Long at WSU) were used for these studies as these cell lines harbor wild type EGFR and express relatively similar protein expression of PAFR [28,38]. The A549 cell line was cultured in F-12K medium with 10% FBS, 2.5 mL antibiotic-antimycotic, 2.5 mL penicillin-streptomycin, and 15 µL of 2M magnesium chloride. H1299 cells were cultured in RPMI-1640 medium with 10% fetal bovine serum, 2.5 mL antibiotic-antimycotic, 2.5 mL penicillin-streptomycin, 2.25 mL of 40% glucose, and 5 mL of 100 mM sodium pyruvate. These cell lines were maintained at 37 °C with 5% CO2 and 95% humidity.

### 4.3. Measurement of ROS Generation

The assessment of ROS generation was done by the ROS detection assay kit, which is based on widely-used H2DCFDA methodology. According to the kit’s manual protocol, A549 cells were seeded in 96 well plates, and cultured overnight. The media was removed and cells were washed with 100 μL ROS assay buffer followed by the addition of 100 μL of 1X ROS label per well, diluted in ROS assay buffer, and incubated for 45 min at 37 °C in the dark. After that, the ROS label was removed, and a 100 μL ROS assay buffer was added, and cells were then treated with 0.1% DMSO or various doses of gefitinib and erlotinib (25, 50, and 75 µM in 100 μL), and incubated for 30 min. Cells containing only 100 μL ROS assay buffer were used as an additional control, cells with only 100 μL of 1X ROS label were used as a negative control, and cells with 100 μL of 1X ROS inducer served as a positive control. In separate experiments, the effects of antioxidant, N-acetylcysteine (NAC), and PAFR antagonist, WEB2086 were evaluated on targeted therapies, and CPAF and PMA-induced ROS generation. The fluorescence was measured at Ex/Em = 495/529 using a Synergy microplate reader (BioTek, Winooski, VT, USA) at Proteome Analysis Laboratory (PAL) core facilities.

### 4.4. Cell Survival Assay

Cell survival was measured by the sulforhodamine-B (SRB) assay as per our previous report [54]. The A549 and/or H1299 cell lines were seeded in 96-well plate at 5 × 10^3^ cell density per well with 200 μL of 1% serum media and following treatments with 0.1% DMSO as vehicle control, and 50 µM concentration of erlotinib and gefitinib in 1% serum media. After 72 h, cells were fixed by adding 100 μL of 20% trichloroacetic acid (TCA) followed by 1-h incubation at 4 °C. Then cells were washed thrice with distilled water and stained with 100 μL 0.4% (weight/volume) SRB (dissolved in 1% acetic acid) followed by 15 min incubation in dark at room temperature. Unbound dye was removed by three washes with 1% acetic acid followed by air drying. Finally, the protein-bound dye was dissolved by 150 μL of 10 mM unbuffered Tris base [tris (hydroxymethyl) aminomethane] for 10 min on a shaker, and absorbance was read at 570 nm with the Synergy H1Mf plate reader. Treatment groups were normalized with the control (0.1% DMSO) group.

### 4.5. Apoptosis Assay

The apoptosis induction was assessed by quantitative caspase 3/7 activity assay using kit’s manual protocol, as described by us previously [73]. For this, A549 cells were seeded overnight into 6-well plates followed by treatments with 0.1% DMSO or erlotinib or gefitinib (50 μM). After 4 and 72 h of incubation, cells were homogenized in hypotonic extraction buffer (HEB) and 0.5 mg of total extracted protein was incubated with Caspase-3/7 Glo reagent. Finally, caspase 3/7 activity was measured using Synergy H1 Luminescence microplate reader (BioTek, Winooski, VT, USA) at Proteome Analysis Laboratory (PAL) core facilities.

### 4.6. siRNA Transfection, RNA Extraction and Quantitative Real-Time PCR (qRT-PCR) Analysis

The A549 cells were transfected using lipofectamine 3000 with three separate clones (i.e., transcripts) of PAFR siRNA as per the manufacturer’s protocol. To optimize the condition, following transfection PAFR knockdown was evaluated at 24 and 48 hours’ time points by analyzing the relative PAFR mRNA expression. For this, the total RNA was extracted from A549 cells by TRIzol extraction method, and extracted RNA was quantified with Nanodrop One spectrophotometer (Thermo Fisher Scientific, Waltham, MA, USA). High-Capacity cDNA Reverse Transcription kit was used to transcribed RNA samples to cDNA for the analysis of the PAFR mRNA expression using an SYBR green-based, quantitative fluorescent PCR method as per our previous reports [34,66]. The fluorescence was detected using a StepOne Real-Time PCR machine (Applied Biosystems, Foster City, CA, USA). The primers used in this experiment were specific for PAFR, and GAPDH was used as an endogenous control to normalize PAFR expression. The quantification of each PCR product was analyzed using the 2^−∆∆Ct^ method.

### 4.7. Microvesicle Particles (MVP) Extraction and Analysis

The extraction and analysis of MVP were done from the culture supernatants of NSCLC cell lines as per our previous reports [34,35,36]. In brief, A549 and H1299 cell lines were cultured overnight or until cells reach 80% to 90% confluency followed by washing the cells thrice with HBSS (no phenol red) only. Then cells were treated with 0.1% EOH and DMSO for negative controls, 100 nM CPAF and PMA for positive controls, and gefitinib and erlotinib at various concentrations (25, 50, and 75 µM) in HBSS containing 1% BSA. After incubations at various time points (1, 2, 4, 6, 8, 12, and 16 h) for initial experiments, and for 4 h for the rest of the experiments, the supernatants were collected and centrifuged at 2000× *g* for 20 min at 4 °C to remove cells, and cell debris. Then, the supernatants were collected and centrifuged the cell-free solution again at 20,000× *g* for 70 min at 4 °C. The pelleted MVPs were resuspended with 100 μL of filtered PBS and used for the nanoparticle tracking analysis (NTA). The size and concentration of these MVPs were detected by using a NanoSight 300 (NS300) instrument (Malvern Instruments, UK). MVPs counts were normalized with the cell count of each treatment [34,35,36]. Similarly, the effects of PAFR knockdown, aSMase inhibition, and MAPK pathways inhibitors on targeted therapies-induced MVP release were analyzed.

### 4.8. Statistical Analysis

Statistical analysis was assessed by GraphPad Prism software version 7.0 (GraphPad Software, San Diego, CA, USA). All in vitro experiments were repeated, independently, at least three times. Data were analyzed by Student’s t-test (to compare between two groups) or one-way ANOVA (for more than two groups) with post hoc Tukey or Bonferroni multiple comparison tests. The value of *p* < 0.05 was considered to indicate a statistically significant difference between the tested groups.

## Abbreviations

PAF	Platelet-activating factor
PAFR	Platelet-activating factor-receptor
Ox-GPCs	Oxidized glycerophosphocholines
MVP	Microvesicle particles
ROS	Reactive oxygen species
MAPK	Mitogen-activated protein kinase
STAT3	Signal transduction and activator of transcription
NSCLC	Non-small cell lung cancer
SCLC	Small-cell lung cancer
aSMase	Acid sphingomyelinase
Imip	Imipramine
PMA	Phorbol myristate acetate
CPAF	Carbamoyl-PAF
COX-2	Cyclooxygenase type 2
PAF-AH	PAF-acetyl hydrolase
Tregs	Regulatory T cells
EGFR	Epidermal growth factor receptor
Gef	Gefitinib
Erlo	Erlotinib
ERK PLC PI3K	Extracellular signal-regulated kinase Phospholipase C Phosphoinositide 3-kinase
